# Body Image Relates to Exercise-Induced Antinociception and Mood Changes in Young Adults: A Randomized Longitudinal Exercise Intervention

**DOI:** 10.3390/ijerph17186801

**Published:** 2020-09-18

**Authors:** Angelika Maurer, Sebastian Deckert, Claudia Levenig, Theresa Schörkmaier, Carolin Stangier, Ulrike Attenberger, Monika Hasenbring, Henning Boecker

**Affiliations:** 1 Functional Neuroimaging Group, Clinic for Diagnostic and Interventional Radiology, University Hospital Bonn, 53127 Bonn, Germany; angelika.maurer@ukbonn.de (A.M.); S.Deckert@drk-kliniken-berlin.de (S.D.); 2 Department of Medical Psychology and Medical Sociology, Ruhr University Bochum, 44780 Bochum, Germany; Levenig@medpsych.ruhr-uni-bochum.de (C.L.); Hasenbring@medpsych.ruhr-uni-bochum.de (M.H.); 3 German Center for Neurodegenerative Diseases, 53105 Bonn, Germany; theresa.schoerkmaier@dzne.de (T.S.); carolin.stangier@dzne.de (C.S.); 4 Clinic for Diagnostic and Interventional Radiology, University Hospital Bonn, 53127 Bonn, Germany; ulrike.attenberger@ukbonn.de

**Keywords:** body image, exercise, pain, physical efficacy, mood

## Abstract

*Background:* An important motivation for adolescents and young adults to engage in aerobic exercise (AE) is to improve fitness, body composition and physical appearance. These parameters have an impact on bodily perception as conceptualized by the ‘body image’ (BI) construct. AE is known to have positive effects on pain perception, mood, and body image (BI). However, no study has hitherto investigated their interrelationship within one study. *Methods:* Participants were randomly assigned to an intervention group (IG, *n* = 16, 6 months of AE) or a passive control group (CG, *n* = 10). Frankfurt Body-Concept Scales (FKKS), Positive and Negative Affect Scale (PANAS), State and Trait Anxiety Inventory, warmth and heat pain thresholds (WPT, HPT), pain tolerance, and graded exercise test data from baseline (T0) and the end of the intervention (T6) were analyzed using a paired *t*-test (*p* < 0.05). *Results:* A significant increase in the BI dimension ‘physical efficacy’ was identified from T0 to T6, which correlated positively with PANAS Positive Affect Scale and HPT. *Conclusion:* Data in young adults undergoing AE indicate that changes in the BI sub-category ‘physical efficacy’ are closely linked with changes in positive affect and antinociception. These novel findings suggest that BI plays a role in antinociception and positive affect.

## 1. Introduction

Aerobic exercise (AE), either voluntary [1] or prescribed [2], has multifaceted effects on physiological and psychological well-being throughout the life-span, i.e., from childhood [3] to older adults [4,5]. An important motivation for adolescents and young adults to engage in aerobic exercise (AE) is to improve fitness, body composition and physical appearance [6,7], all of which are parameters that have an impact on bodily perception as conceptualized by the ‘body image’ (BI) construct. The BI definition covers perceptions, thoughts, feelings, and attitudes an individual has towards the own body [8,9] and this definition includes appearance aspects [10], individual perception of body functionality [11], perception of self-esteem, body shape and fitness [12,13,14]. While studies have mainly focused on the perceptual aspects of BI in association with, e.g., eating disorders or pain [15,16,17], recently more attention was given to the attitudinal dimensions of BI like evaluative-affective and cognitive-behavioral aspects [8].

Regular AE has been shown to go along with small to moderate positive effects on BI [10,18,19,20,21,22] and individuals seem to benefit to a larger extent from endurance as compared to strength training [23]. The relationship between physical fitness and BI seems to be mutually bidirectional: physical activity is thought to result in positive BI [21] and, likewise, individuals with positive BI appear to be motivated to stay physically active [8,24]. As a consequence, Campbell and Hausenblas [10] pointed towards exercise as a promising intervention for improving negative BI. Their meta-analysis, derived from 57 publications on longitudinal exercise intervention studies, revealed a small effect size for improved BI changes from pre- to post-intervention, as compared to a non-exercise control condition [10].

Research distinguishes between the independent concepts of positive and negative body image [20], which may both exist coincidently. A positive BI is associated with positive mood [20,25] and individuals with a positive BI show more positive well-being and more self-protective attitudes and behaviors [25,26]. Acceptance of one’s body, as conceptualized in the BI construct, also impacts on bodily deficits and the subjective perception of body functionality [20]. Previous research has provided ample evidence that AE can improve mood, ranging from more general states of positive mood [27,28,29] and increased stress resistance [30], along with specific anxiolytic [31] or anti-depressive effects [32].

Moreover, AE has positive effects on pain perception. Exercise-induced hypoalgesia (EIH), is a well-established psychophysical phenomenon that is thought to play a role in prevention and non-pharmacological management of chronic pain states. Experimentally, EIH can be elicited as acute local and/or remote heat or cold pain threshold changes on sensory testing, or by enhanced pain tolerance [33,34,35,36]. Beyond short-term EIH during and shortly after acute bouts of exercise, there is human evidence in favor of long-term modulatory down-regulation of pain perception following repeated AE. For example, elevated pain tolerance has been identified in athletes as compared to normally active control subjects [37]. Meta-analytical analyses have shown that effects of exercise on pain tolerance are of moderate to large effect size, whereas available data on pain thresholds appear to be less uniform [38]. Biological as well as psychological theories have been put forward as mechanisms underlying EIH, often linking reduced pain perception with associated positive mood changes [39].

Whether the biological and psychological concepts are mutually exclusive or rather interrelated remains unknown as no study, we are currently aware of, has measured the three components (antinociception, mood and BI) within one single study design. Therefore, the purpose of the present study was to determine whether changes in BI concept predict exercise-induced changes in mood and pain perception and, if so, to unravel which subcomponent of the BI concept—‘self-acceptance of one’s body’, ‘physical efficacy’, or ‘health’—has a driving force. Hypothesizing differential downstream behavioral effects depending on (i) long-term PE (intervention group) or (ii) continued sedentary behavior (control group), our analyses of the BI questionnaire [40], were meant to decipher which ‘state of mind’ is related to the affective and antinociceptive effects of regular exercise. Notably, the FKKS covers cognitive and affective components of the BI, in contrast to many other studies emphasizing merely perceptual components [15]. This is the first study exploring these three dimensions of BI on affective and antinociceptive outcomes in a randomized longitudinal intervention. Previous work demonstrated ‘physical efficacy’ to be the strongest factor determining negative BI in patients suffering from subacute pain, as compared to the healthy controls [41], hence, providing a specific hypothesis for this study.

## 2. Materials and Methods

### 2.1. Experimental Design

**Figure 1:**
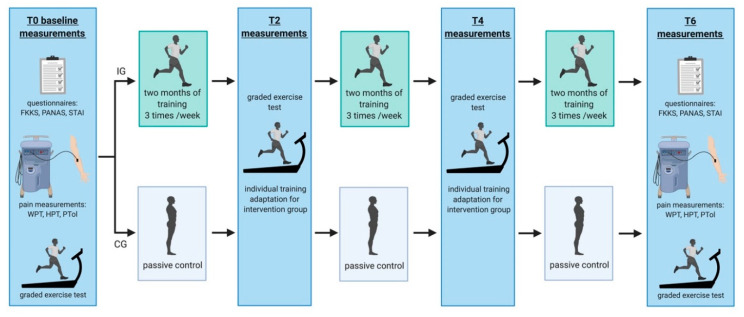
Study design. CG = control group, FKKS = Frankfurt Body-Concept Scales, HPT = heat pain threshold, IG = intervention group, PANAS = Positive and Negative Affect Schedule, PTol = pain tolerance, STAI = State and Trait Anxiety Inventory, WPT = warmth perception threshold.

‘RUNSTUD’ is a longitudinal AE intervention study of six months duration in which young (24.2 ± 4.2 years) sedentary individuals were pseudo-randomly assigned to a physical intervention group (IG: 3 × per week aerobic exercise) or a control group (CG: no regular aerobic exercise). Prior to inclusion in the study, all participants underwent a medical healthcare check involving a short anamnestic questionnaire, auscultation of the lung and heart, and a 12-channel resting electro cardiogram to exclude major physical health risks for subsequent exercise tests and trainings. Subsequently, all participants (IC and CG) performed a graded exercise test (GXT) on a treadmill to determine their individual fitness level. During the study, participants completed this test four times in total: at baseline (prior to the intervention, T0), after 2 months (T2), 4 months (T4) and 6 months (T6). Parallel to the GXT, participants underwent additional examinations every 2nd months including: MRI (3T and 7T), neuropsychological tests, and blood sampling for epigenetics which are not the focus of the current study. The current work is a purely cognitive and affective behavioral sub-analysis of the data derived from RUNSTUD, focusing on pain perception/pain tolerance, mood and BI, and their interaction dependent on long-term exercise or continued sedentary behavior. The body image questionnaire was acquired exclusively at baseline and at the end of the study (after 6 months; T6), hence, restricting the analyses to these two time-points. The study design is shown in Figure 1.

### 2.2. Participants

Participants were recruited in and around Bonn, Germany via social media, announcements and flyers. Due to the expectation of higher dropout rates in the IG, participants were allocated to the groups with a ratio of 2:1 (2 intervention:1 control). 59 participants with a sedentary lifestyle were initially included in the study, 27 of whom dropped out due to loss of interest and lack of time during the course of the study. Of the remaining 32 participants, complete datasets for this study design were collected only in *n* = 26 (13 males and 13 females). Participants were without any prior history of neurological or psychiatric illness, orthopedic or general health problems that may have prohibited physical activity. From a set of questionnaires (socio-demographics, Edinburgh Handedness Inventory [42]; German vocabulary test [43]) descriptive characteristics including age, education, verbal intelligence level and handedness of participants were acquired. Furthermore, psychiatric questionnaires were administered, including the Mini International Neuropsychiatric Interview (MINI, German Version 5.0.0, Psychiatrische Universitätsklinik München, Munich, Germany) [44], the State and Trait Anxiety Inventory (STAI) [45] and the Beck Depression Inventory (BDI) [46]. One of the 26 participants showed a slightly elevated BDI score of 11, but veritable depression could be ruled out by a physician’s examination. Participants’ characteristics are summarized in Table 1. All participants were informed about the purpose of the study and the possible risks involved before providing written informed consent to participate. The study was approved by the local ethics committee and conducted according to the Declaration of Helsinki at the University Hospital Bonn (Ethikkommission an der Medizinischen Fakultät der Rheinischen Friedrich-Wilhelms-Universität Bonn: Nr. 370/15).

### 2.3. Graded Exercise Test

All participants performed a GXT on a treadmill (Woodway GmbH, Weil am Rhein, Germany) with an initial running speed of 6 km/h, 1% incline and 1∙km/h increments every 3 min to determine maximum oxygen uptake (VO_2max_) and maximum heart rate (HR_max_). Exhaustion was considered with the attainment of at least two of the following criteria: high levels of blood lactate (BLa; 8–10 mmol/L); a perceived rate of exertion of ≥ 18 and/or a heart rate (HR) of ±10 bpm of age-predicted maximum (220-age) [47]. VO_2_ (Cortex meta-analyzer 3B, Leipzig, Germany) and HR (Polar Electro, Kempele, Finland) were averaged over the last 30 s of each step, and capillary blood lactate samples were taken from the fingertip in the last 15 s of each step. To determine blood lactate concentration, 20 µL of capillary blood were immediately mixed with 1 mL hemolysis solution and analysed via an amperometric-enzymatically procedure (Biosen C_line, EKF Diagnostic Sales, Magdeburg, Germany). At the same time-points, rating of perceived exertion (RPE) was assessed using the 6 (no exertion at all/extremely light) to 20 (maximal exertion) points Borg scale [48]. Participants were not allowed to perform strenuous exercise 48 h prior the testing and were instructed to refrain from caffeine 6 h prior to exercise testing.

### 2.4. Endurance Exercise Intervention

Throughout the 6-months intervention phase participants of the intervention group completed two supervised running sessions per week on the treadmill in the laboratory and one at home in a flat terrain of 25 to 45 min each, resulting in a total of 78 prescribed training sessions, of which 62.8 ± 9.1 (80.5 ± 11.7%) were performed on average. An activity tracker was used to monitor participants’ heart rate, steps and distance of each home training as well as general daily physical activities.

The endurance exercise of the running intervention group was designed as extensive interval training with three to five minutes intervals at 75–80% of HR_max_ and three to five minutes active recovery with six to eight repetitions. To ensure a steady progression of physical adaptations, exercise intensity was adapted individually according to the results of each GXT (2 and 4 months after baseline). Lactate thresholds were determined using the modified method of [49] (D_max_). Participants in the control group were instructed to maintain their usual lifestyle, to refrain from any kind of exercise and to continue their normal dietary and physical activity practices throughout the study. Both groups (IG and CG) were asked to wear a fitness tracker and to record every physical activity with the tracker.

### 2.5. Frankfurt Body-Concept Scales (FKKS)

The Frankfurt Body Concept Scales (German: Frankfurter Körperkonzeptskalen, FKKS) [40] is a self-rating instrument that assesses cognitive-affective aspects of the individual body image. It consists of nine subscales, of which three subscales were used in the present study: The scale ‘physical efficacy’ (SKEF, 10 items, e.g., ‘I do a lot of sports’) assesses individual attitudes with regard to fitness. The scale ‘health’ (SGKB, 6 items, e.g., ‘I feel healthy’) assesses the individual perception of the own state of health. The scale ‘self-acceptance of one’s body’ (SSAK, 6 items, e.g., ‘I am confident with my appearance’) assesses individual attitudes related to concerns of attractiveness.

All items were rated by a 6-point-Likert-scale ranging from ‘very much’ to ‘not at all’. The higher the score the more positive the body image can be interpreted.

### 2.6. Positive and Negative Affect Schedule (PANAS)

The Positive and Negative Affect Schedule is one of the most widely used self-report questionnaires for affective measures [50]. It consists of 20 items: 10 for the Positive Affect scale (e.g., interested, excited), 10 for the Negative Affect scale (e.g., distressed, upset). Each item is rated on a 5-point scale ranging from 1 (not at all) to 5 (very much).

### 2.7. Determination of Sensory and Heat Pain Threshold

Measurements of warmth perception thresholds (WPT) and thermal heat pain thresholds (HPT) were performed pre and post intervention using a MEDOC-TSA II thermal pain stimulation device (Medoc, Ramat-Yishai, Israel). Starting at a temperature of 32 °C, the ‘ascending methods of limits’ option with a temperature increase of 0.5 °C per second was used. Therefore, a 9 cm^2^ contact thermode was placed at the non-dominant left volar forearm 2 cm below the arm bend. For the WPT, participants were instructed to press a button with their right hand as soon as experiencing a ‘temperature change’. For the HPT, they were instructed to press a button when the thermal heat perception was accompanied for the first time by a ‘painful sensation’. Three test trials were followed by 5 main trials [51]. The test trials allowed the participants to familiarize with the protocol and were not included in the data analysis. The 5 main trials were averaged to determine the respective WPT and HPT. When determining the thresholds, the WPT was always acquired first, followed by the determination of the HPT.

### 2.8. Pain Tolerance Determination

To determine pain tolerance (PTol) as a measure of EIH pre and post intervention, participants were asked to place their left hand in 2 °C cold water using a commercially available refrigerated circulating bath (temperature stability ± 0.01 °C; AD15R-30-V12V, VWR International, Radnor, PA, USA). The instructions for the cold pressor test (CPT) were to withdraw the hand from the cold water as soon as perceived pain was no longer tolerable. The elapsed time was measured. For safety reasons the maximal time of testing was set at 4 min. During the test, participants were requested to indicate perceived pain on a VAS (pain intensity) every 20 s. Withdrawal was expected to occur at a maximum pain intensity level on the VAS, thus, validating the PTol measurements. PTol was always determined directly after WPT and HPT.

### 2.9. Statistical Analysis

All statistical analyses were performed using SPSS 25 (SPSS Inc., Chicago, IL, USA). Normal distribution was verified by a Shapiro–Wilk test. Differences were considered significant at *p* ≤ 0.05. Additionally, correlation coefficient ‘r’ as an effect size will be reported, with r < 0.30 representing a small effect, 0.3 < r < 0.5 a medium effect, and r > 0.5 a large effect.

### 2.10. Within-Group Analyses

Significant differences between T0 and T6 of the physiological data, the data of the questionnaires (body image, anxiety, mood), and the data of the pain measurements were statistically analyzed using either a paired *t*-test (for normally distributed data) or a Wilcoxon rank-sum test (for not normally distributed data).

### 2.11. Between-Group Analyses

To test for group differences, individual ‘T6–T0’ deltas (Δ) were created for all acquired data and entered in either an independent *t*-test or a Mann–Whitney U test.

### 2.12. Correlation Analyses

To investigate whether the dimensions of the FKKS (that show a significant effect due to the intervention) correlate with individual fitness level (indexed as speed at D_max_ in the GXT), mood (PANAS), anxiety (STAI), or pain perception (indexed as WPT, HPT, PTol), additional one-tailed correlation analyses were performed using the deltas of T6 minus T0.

In the current study, speed at D_max_ has been chosen for correlation analyses, as the speed at lactate threshold is a good physiological parameter to detect changes in endurance performance. This has been supported by previous studies [52] that showed that the speed at the lactate threshold integrates V̇O_2max_, %V̇O_2max_, and running economy and therefore explains differences in aerobic endurance performance better than by VO_2max_ alone. In addition, due to artifacts in the recording, the VO_2max_ values could not be determined for two participants of the sample.

## 3. Results

### 3.1. Physiological Data

**Figure 2:**
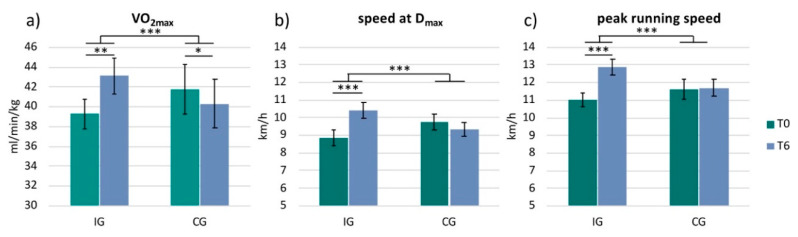
Physiological parameters. Bar plots show the physiological parameters for each group (IG = intervention group and CG = control group) and for each timepoint (T0 and T6): (**a**) VO_2max_; (**b**) speed at D_max_; (**c**) peak running speed; *** *p* < 0.001, ** *p* < 0.01, * *p* < 0.05.

The physiological data were not normally distributed. Therefore, we applied the Wilcoxon rank-sum test and the Mann–Whitney U test. Due to artifacts, the VO_2max_ values of two participants in the IG could not be determined. These participants were therefore excluded from the analysis of the VO_2max_ data (*n* = 14). Analysis revealed a significant increase in VO_2max_ in the IG from 39.3 ± 5.3 (mean ± standard deviation) to 43.1 ± 6.4 mL/min/kg (z = −3.30; *p* = 0.001; r = −0.88). The CG showed a significant decrease in VO_2max_ from 41.7 ± 7.5 to 40.3 ± 7.4 mL/min/kg (z = −1.99; *p* = 0.047; r = −0.63) (Figure 2a). Analysis also revealed a significant increase in running speed at D_max_ in the IG from 8.9 ± 1.8 to 10.4 ± 1.7 km/h (z = −3.52; *p* < 0.001; r = −0.88) (Figure 2b) and peak running speed from 11.0 ± 1.6 to 12.9 ± 1.7 km/h (z = −3.62; *p* < 0.001; r = −0.91) (Figure 2c). The CG did not show any significant changes in these two parameters (D_max_: z = −0.77; *p* = 0.441; r = −0.24; peak running speed: z = −0.38; *p* = 0.705; r = −0.12). Comparing the two groups with each other (IG versus CG) revealed a significant difference between the groups for all three parameters: VO_2max_ (IG: increase by 3.8 ± 2.7 mL/min/kg, CG: decrease by −1.4 ± 2.0 mL/min/kg; U = 4.5, z = −3.84, *p* < 0.001, r = −0.78), speed at D_max_ (IG: 1.6 ± 1.1 km/h, CG: −0.4 ± 1.2 km/h; U = 6.0, z = −3.90, *p* < 0.001, r = −0.77) and peak running speed (IG: 1.9 ± 0.6 km/h, CG: 0.1 ± 0.9 km/h; U = 11.0, z = −3.82, *p* < 0.001, r = −0.75) (Figure 2).

### 3.2. Frankfurt Body-Concept Scales

**Figure 3:**
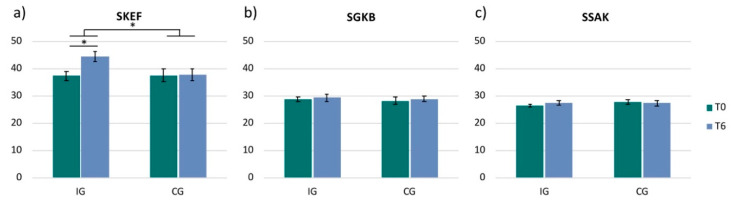
Frankfurt Body-Concept Scales. Bar plots show the 3 dimensions of the questionnaire for each group (IG = intervention group and CG = control group) and for each timepoint (T0 and T6): (**a**) ‘physical efficacy’ (SKEF); (**b**) ‘health’ (SGKB); (**c**) ‘self-acceptance of one’s body’ (SSAK); *p* < 0.05.

The analysis of the three dimensions (SKEF, SSAK, SGKB) of the FKKS only revealed a significant change in the dimension ‘physical efficacy’ (SKEF). The IG showed a significant increase in SKEF from 37.3 ± 6.2 to 44.6 ± 7.1 (t(15) = −4.12, *p* = 0.001, r = 0.73), while the CG did not reveal any changes in this dimension (T0: 37.6 ± 7.0; T6: 37.7 ± 6.7; t(9) = −0.11; *p* = 0.913, r = 0.04) (Figure 3a). Comparing the two groups with each other (IG versus CG) revealed a significant difference between the groups in the SKEF (IG: 7.3 ± 7.0, CG: 0.1 ± 2.8; t(24) = 3.04, *p* = 0.006, r = 0.53) (Figure 3a).

The dimensions ‘health’ (IG: t(15) = −0.56, *p* = 0.585, r = 0.14; CG: t(9) = −0.53, *p* = 0.611, r = 0.17*)* and ‘self-acceptance of one’s body’ (IG: z = −1.72; *p* = 0.086; r = −0.43; CG: z = −0.60; *p* = 0.546; r = −0.19) did not reveal any significant changes in any of the two groups or between groups (SGKB: t(24) = 0.05, *p* = 0.962, r = 0.01; SSAK: U = 52.0, z = −1.50, *p* = 0.133, r = −0.29) (Figure 3b,c).

### 3.3. Mood and Anxiety Scales

**Figure 4:**
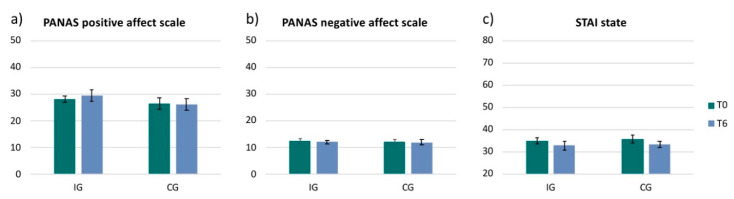
PANAS and STAI. Bar plots show the 2 dimensions of the PANAS (positive and negative) and the STAI state for each group (IG = intervention group and CG = control group) and for each timepoint (T0 and T6): (**a**) PANAS Positive Affect Scale; (**b**) PANAS negative affect scale; (**c**) STAI state.

The PANAS Positive Affect Scale revealed a small increase of positive mood from T0 to T6 in the IG (2.3 ± 6.5; t(15) = −0.90, *p* = 0.385, r = 0.23) and no change in the CG (−0.2 ± 9.0; t(9) = −0.07, *p* = 0.946, r = 0.02) (Figure 4a). The PANAS negative scale showed no change in either of the groups (IG: −0.3 ± 3.1, t(15) = 0.40, *p* = 0.696, r = 0.10; CG: −0.3 ± 2.9, t(9) = −0.33, *p* = 0.752, r = 0.11) (Figure 4b). The acquired data of the STAI state showed a decrease in anxiety in both groups (IG: −2.2 ± 4.2, z = −1.77; *p* = 0.077; r = −0.44; CG: −2.4 ± 6.0, z = −1.55; *p* = 0.122; r = −0.49) (Figure 4c). However, both questionnaires, the PANAS (positive scale and negative scale) and the STAI state did not reveal any significant changes from T0 to T6 measurements in either of the groups and no effect between groups (PANAS positive: t(24) = −0.81, *p* = 0.428, r = 0.16; PANAS negative: t(24) = −0.01, *p* = 0.991, r = 0.00 ; STAI state: z = −0.77; *p* = 0.442; r = −0.15) (Figure 4).

### 3.4. Pain Thresholds and Pain Tolerance

**Figure 5:**
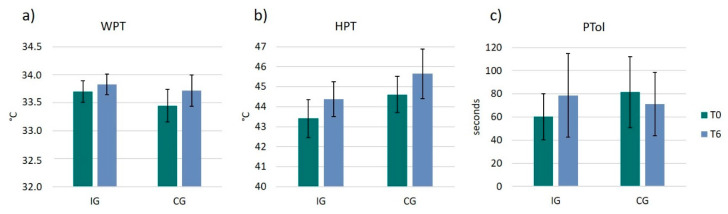
Pain perception and pain tolerance. Bar plots show the 3 parameters of pain perception and pain tolerance for each group (IG and CG) and for each timepoint (T0 and T6): (**a**) warmth perception threshold (WPT); (**b**) heat pain threshold (HPT); (**c**) pain tolerance (PTol).

WPT and HPT showed both an increase in the IG (WPT: 0.1 ± 0.6 °C, t(14) = −0.85, *p* = 0.412, r = 0.22; HPT: 1.3 ± 3.2 °C, t(15) = −1.33, *p* = 0.203, r = 0.32) and the CG (WPT: 0.3 ± 0.6 °C, t(8) = −1.45, *p* = 0.187, r = 0.46; HPT: 1.0 ± 1.5 °C, t(8) = −1.98, *p* = 0083, r = 0.57) (Figure 5a,b). However, none of these two pain measurements (WPT, HPT) revealed any significant changes from T0 to T6 or between groups.

For the statistical analysis of pain tolerance participants were excluded who were able to hold their hands in the cold water for 4 min already at the beginning of the study (ceiling effect). Unfortunately, the final group size for pain tolerance included only *n* = 20 participants (IG: *n* = 14; CG: *n* = 6), therefore, the statistical analysis for the between group comparison of PTol does lack statistical power and robustness. However, to provide a complete picture of the data, we will still describe the results. While there was an increase in PTol in the IG from 60.2 ± 48.9 to 78.6 ± 88.4 s (z = −0.22; *p* = 0.826; r = −0.06), the CG group showed a decrease in PTol from 81.5 ± 53.1 to 71.0 ± 47.5 s (z = −0.94; *p* = 0.345; r = −0.38). However, there was no significant difference between groups (U = 2900; z = −1.07; *p* = 0.283; r = −0.24) (Figure 5c).

### 3.5. Correlation Analyses

**Figure 6:**
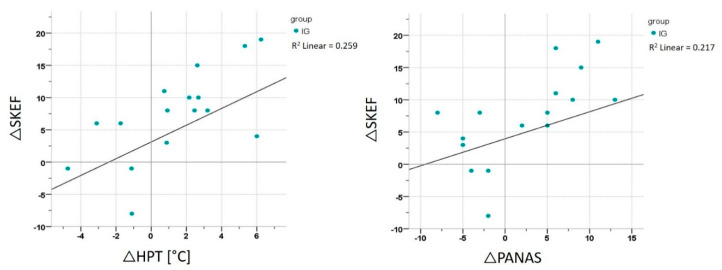
Correlation analysis IG. Correlation between the SKEF and the PANAS Positive Affect Scale and HPT for the IG (intervention group).

Correlation analyses were performed for both groups separately and for all participants together, using the Δ of T6 minus T0. The correlation analyses were performed between the ΔSKEF and the Δspeed at D_max_, ΔPANAS, ΔSTAI trait, ΔWPT, ΔHPT and ΔPTol. Analyses revealed a significant correlation in the IG between the ΔSKEF and ΔPANAS Positive Affect Scale (r = 0.655, *p* = 0.006) and between the ΔSKEF and ΔHPT (r = 0.659, *p* = 0.006) (Figure 6). However, there was no significant correlation between ΔSKEF and the fitness parameter Δspeed at D_max_. The CG did not reveal any significant correlations.

**Figure 7:**
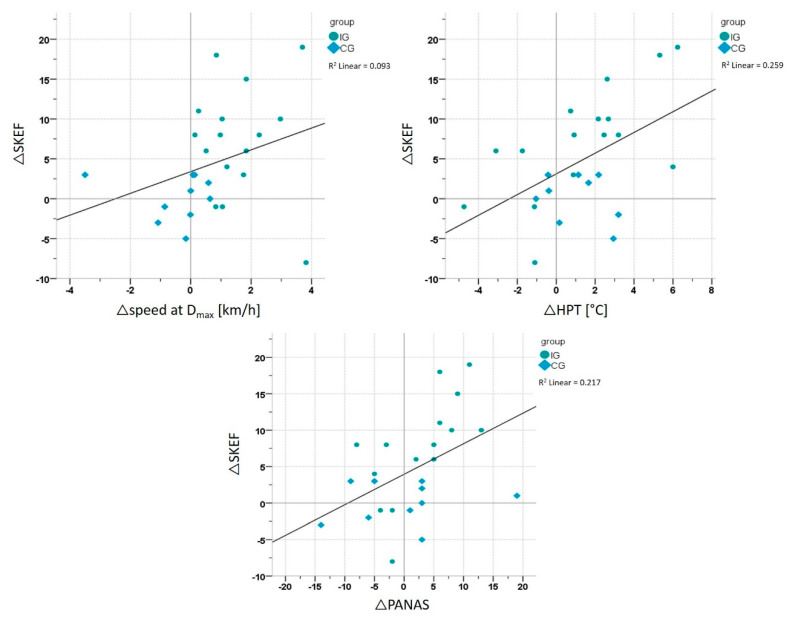
Correlation analysis whole sample. Correlation between the SKEF (‘physical efficacy’) and speed at D_max_, the PANAS Positive Affect Scale and HPT (heat pain threshold) for both groups (IG = intervention group and CG = control group) together.

Correlation analyses including both groups (IG and CG) revealed a significant correlation between the ΔSKEF and Δspeed at D_max_ (r = 0.443, *p* = 0.023; *n* = 26), ΔSKEF and ΔPANAS Positive Affect Scale (r = 0.466, *p* = 0.016; *n* = 26) and the ΔSKEF and ΔHPT (r = 0.509, *p* = 0.009; *n* = 25) (Figure 7).

## 4. Discussion

This randomized six months AE-intervention in young healthy sedentary individuals sheds new light on the BI changes occurring in the context of long-term exercise and associated fitness adaptations: For the first time, we demonstrate AE-induced (sub-category) BI-changes and their link to well-established psychophysical phenomena seen after long-term exercise.

Regular PE in the intervention group (but not in the control group) was associated with expected fitness increases; these were evidenced by significant improvements in VO_2max_, running speed at D_max_, and peak running speed, hence, providing proof of the efficacy of the PE intervention. While mood scales, anxiety scales and pain indices (i.e., WPT, HPT, and PTol) revealed no significant change over time in the intervention group, the FKKS revealed a significant sub-category BI-change in the dimension SKEF, supporting previous cross-sectional data related to exercise (see discussion below). The observed changes in the SKEF hint towards AE-induced improvements in ‘physical efficacy’, i.e., sensed and experienced bodily strength, skillfulness, looseness, and flexibility [40], rather than in other BI sub-components like ‘health’ (i.e., perception of the own state of health) or ‘self-acceptance of one’s body’ (i.e., individual attitude related to concerns of attractiveness). Correlation analysis showed that the SKEF changes, tested in the entire study population as well as in the intervention group alone, were significantly related to the HPT changes and mood changes in the positive affect scale. Correlation analyses thus support the study hypothesis of ‘physical efficacy’ being the strongest BI-predictor for pain modulation, and now further extending its role also for positive mood changes.

Our findings nicely align with previous observations indicating regular AE to have long-term modulatory effects on both, pain and mood: Antinociceptive effects are increasingly acknowledged as long-term effects of regular AE training, with meta-analytical analyses reporting pain threshold elevations [38], as in our study. There is, however, a gap in research focusing on pain processing and BI: Osumi et al. [53] found lower heat pain thresholds in participants with lower BI and concluded that negatively evaluated body appearance is associated with increased pain sensitivity. On the other hand, participants with a more positive body image are expected to have higher pain thresholds, an assumption that is also supported by the positive correlation between SKEF and HPT in the present study. Previous work has shown that the SKEF, in comparison to other components of the FKKS, is most closely related to pain states [41]: Patients with chronic or subacute pain states have a more negative body image of ‘physical efficacy’, as compared to healthy controls [41]. The current longitudinal data extend these cross-sectional findings in patients, by showing that AE modulates precisely the ‘physical efficacy’ dimension of the BI concept.

Regarding mood, it has been reported that people undergoing regular AE training have higher levels of mental well-being [54,55] and lower incidences of depression [56]. AE was shown to be ‘moderately more effective’ than a control intervention in reducing depression symptoms [57]. Randomized controlled trials (RCTs) support AE to be effective as treatment (alone or as an adjunct intervention to antidepressant medication) for unipolar depression [58]. Considering BI, there is evidence that people revealing a positive BI have lower scores of depression and more well-being [25]. Our correlation analysis suggests that BI improvements in the sub-category ‘physical efficacy’ after AE are related to positive mood.

Self-efficacy is known to influence different health behaviors [59] and it was suggested ‘that self-efficacy is one of a number of potential mediators of the effects of physical activity’ [60] on different psychological outcomes (e.g., depression, mood, pain, etc.). Our findings therefore suggest that protective antinociceptive and mood-stabilizing effects of PE are linked with reinforcing self-efficacy. Higher self-reported efficacy appears to go along with higher levels of physical activity, which is referred to as the ‘self-efficacy’s reciprocal relationship with physical activity’ [60]. Activity counselling related to self-efficacy was shown to increase physical activity, in turn increasing self-efficacy for physical activity [61]. Accordingly, we would like to speculate that higher SKEF associated with AE may also play a role for maintaining and reinforcing protective and preventative mechanisms relevant for affective and pain regulation. Preventive behavioral gains could be operationalized via ‘social cognitive theory’ (SCT), where self-efficacy, as one of many different psychological constructs, has been ‘consistently associated with physical activity, function, and well-being’ [60]. As concrete example related to pain, self-efficacy was associated with clinical outcomes and self-ratings in a prospective trial examining the progression of disability in older adults with knee pain [62]: Participants who had low self-efficacy and low strength at baseline had the largest 30-month decline in these outcomes [62]. Support for the role of self-efficacy on affective states comes from experimental manipulations of self-efficacy [63]: Participants randomly assigned to a high-or low-efficacy condition show different affective responses after a single bout of AE, i.e., ‘with participants in the high-efficacy group reporting more positive and less negative affect than did the low-efficacy group’ [60].

Although our findings were centered on exercise-induced changes in ‘physical efficacy’, corroborating previous findings in chronic pain patients [41] we have to acknowledge that the observation of no significant changes in ‘health’ and ‘self-acceptance of one’s body’ is not in accord with the entire previous research literature [64,65]. We may speculate, however, that no changes in the individually perceived health status had to be expected in the context of this study, as all participants were healthy and young.

Finally, we would like to point out that our findings certainly do not imply that the observed changes in BI are the exclusive variables influencing pain and mood percepts in AE. Rather, we would like to suggest that neurohumoral changes in the brain induced by AE play an elementary role for shaping perceptual and affective aspects of the BI and the resulting neuropsychological state.

### Limitations

As discussed above, the association between the change in SKEF and both, the increase in PANAS Positive Affect Scale and the increase in HPT, suggests SKEF to have impact on both behavioral categories. However, a causal relation cannot be derived at the current stage and further empirical research would be needed to prove such a hypothesis. The sample size per group is quite small and future studies are needed to investigate bigger sample sizes. Moreover, due to the small sample size we were not able to investigate gender differences.

## 5. Conclusions

The FKKS revealed a significant improvement in the IG in the dimension ‘physical efficacy’, supporting the role of BI changes induced by AE. A significant change in mood, anxiety or pain perception could not be detected in either of the groups. However, exercise-induced changes in BI sub-category ‘physical efficacy’ relate to elevated HPT and increased positive mood. This indicates that higher ‘physical efficacy’ induced by AE is associated with elevated mood and higher heat pain thresholds. While it appears to be generally established in the literature that AE has a positive effect on BI, the interrelationships with mood and pain perception studied here are novel and so far not covered by any research we are aware of. Current data from a randomized exercise intervention in healthy participants thus extend the knowledge on changes in BI sub-categories induced by AE and, notably, its relation to pain perception and mood.

## Figures and Tables

**Table 1:** Participants’ characteristics.

	Intervention Group (*n* = 16)	Control Group (*n* = 10)
Age (years)	24.5 ± 4.3	23.7 ± 4.2
Height (cm)	173.4 ± 12.0	176.9 ± 7.9
Mass (kg)	70.2 ± 15.8	71.2 ± 14.1
EHI	75.2 ± 17.3	79.5 ± 13.3
BMI	23.3 ± 3.8	22.7 ± 3.6
VO_2max_ (mL/min/kg)	39.3 ± 5.3 ^a^	41.7 ± 7.5
Speed at D_max_ (km/h)	8.9 ± 1.9	9.8 ± 1.4
HR_max_ (bpm)	198.5 ± 7.9	200.8 ± 8.5
Peak running speed (km/h)	11.0 ± 1.6	11.5 ± 1.8
Education (years)	16.4 ± 3.2	15.8 ± 3.1 ^a^
GVT	107.3 ± 10.3	107.3 ± 8.8
BDI	2.5 ± 3.1	1.4 ± 1.5
STAI trait	34.8 ± 10.1	31.4 ± 6.1 ^b^

Legend: BDI = Becks Depression Inventory (score ≤ 9: no depression); BMI = body mass index; D_max_ = distance maximum method; EHI = Edinburgh Handedness Inventory; HR_max_ = maximum heart rate; GVT = German vocabulary test; STAI = State Trait Anxiety Inventory (range: 20 = not being afraid up to 80 = maximum intensity of anxiety); VO_2max_ = relative maximum oxygen uptake; ^a^ 2 missing values, ^b^ 1 missing value.
